# The psychopathology of the elderly with multimorbidity. Is an integrated training model feasible?

**DOI:** 10.1192/j.eurpsy.2021.2198

**Published:** 2021-08-13

**Authors:** A. Riolo, U. Albert

**Affiliations:** 1 Department Of Mental Health, ASUGI, Trieste, Italy; 2 Department Of Mental Health, University of Trieste, Trieste, Italy

**Keywords:** psychogeriatrics, training, elderly, multimorbidity

## Abstract

**Introduction:**

The aging of the population implies a greater risk of psychopathological events; at the same time multimorbidity constitutes the rule rather than the exception in the manifestations of the health problems of the elderly. Multimorbidity involves many diagnostic-therapeutic interventions, from general practitioners to neurology, geriatrics, psychiatry but these interventions do not appear integrated with each other.

**Objectives:**

Evaluate the availability of psychogeriatrics training programs to increase the interest and skills of the medical profession on the multimorbidity of the elderly.

**Methods:**

We have conducted a review of the scientific literature on integrated training programs in the field of psychogeriatrics over the past decade on pubmed, comparing the different training models proposed.

**Results:**

A still limited amount of articles on integrated psychogeriatric training have been published although the demand for psychogeriatric care continues to increse. The frail elderly seems to have a connotation in terms of costs rather in terms of care; moreover, everything concerning the elderly is reduced to the organic dimension alone, neglecting psychopathology.

**Conclusions:**

The feasibility of integrated training programs between primary care and specialists such as neurologists, geriatricians, psychiatrists is a priority in the field of psychogeriatrics in consideration of the relevant multimorbidity. It is appropriate both to update knowledge and to review the organizational models of care so that the frailty of the elderly with multimorbidity does not quickly translate into disabilities with high social welfare needs. It is also necessary for generalist psychiatry to return to the value of psychopathology of the elderly.

**Disclosure:**

No significant relationships.

